# Evaluating score distributions in the revised Dutch version of the Childhood Health Assessment Questionnaire

**DOI:** 10.1186/1546-0096-6-14

**Published:** 2008-09-11

**Authors:** Jessika W Ouwerkerk, Philomine A van Pelt, Tim Takken, Paul JM Helders, Janjaap van der Net

**Affiliations:** 1 Utrecht School of Health Sciences, Utrecht University, Utrecht, The Netherlands; 2 Department of Pediatric Rheumatology, Wilhelmina Children's Hospital, University Medical Center Utrecht, Utrecht, The Netherlands; 3 Department of Pediatric Physical Therapy & Exercise Physiology, Wilhelmina Children's Hospital, University Medical Center Utrecht, Utrecht, The Netherlands

## Abstract

**Objectives:**

Evaluating the original, and the revised version of the Dutch Childhood Health Assessment Questionnaire (CHAQ). To explore the effect of different score calculation methods and eight more challenging items as proposed by Lam et al. (2004) on the score distribution in a population of patients with Juvenile Idiopathic Arthritis (JIA).

**Methods:**

Two convenience samples of 59 and 31 children with JIA were studied. Box-and-whisker plots and the Kolmogorov-Smirnov (K-S) one-sample test of normality were used, to explore the score distributions.

**Results:**

The results of this study confirm a ceiling effect when using the original CHAQ-30 with either score calculation method. The original CHAQ with the added eight more challenging items and the "mean" score calculation method, as well as the revised CHAQ showed less ceiling effect.

**Conclusion:**

The original CHAQ-38 with the "mean" score calculation method as well as the revised CHAQ are a possible alternative for future studies. However, there is a need for further prospective studies to improve the CHAQ and to support our findings.

## Introduction

The Childhood Health Assessment Questionnaire (CHAQ) is the most widely utilized functional status measure in paediatric rheumatology today. The CHAQ consists of a disability index (30 items; 8 domains), and a discomfort scale (two visual analogue scales) and can be completed by children as well as their parents/guardians. The CHAQ has shown to be a valid, reliable, and sensitive functional status measure in children with Juvenile Idiopathic Arthritis (JIA) [1]. Over the years the use of the CHAQ has also broadened to other childhood rheumatic conditions [2-5].

Despite its advantages and wide use, the CHAQ suffers from a ceiling effect [6]. Therefore it is difficult to discriminate distinct levels of function at the mild end of the disability continuum and to assess improvement in health for less impaired patients [6]. Lam et al. (2004) tried to influence this ceiling effect by testing different response options (visual analogue scale (CHAQ_VAS_), categorical (CHAQ_Cat_), and choice (CHAQ_Choice_)) and by adding eight more challenging items [6]. Respondents were instructed to compare their capabilities to that of their age peers over the last week. The different response options made it possible to asses not only patient's limitations (original CHAQ), but also the patient's strengths. Lam et al. added more challenging items so as to allow less impaired patients to score below the ceiling [4]. The results showed greater sensitivity, a more normal distribution, and a diminished ceiling effect for all three response options. The CHAQ_Cat _showed best concordance as a proxy report and might be easiest to complete.

The scoring rules applied to the original CHAQ are rather complex. The thirty items of the disability index assess eight domains of physical function. Of each domain three components are evaluated: (a) difficulty to perform each activity (0 = no difficulty, 1 = some difficulty, 2 = much difficulty, 3 = unable to do), (b) use of special aids or devices, and (c) required need for assistance of another person. The total score is the average of the highest score in each domain. If component b or c is scored, the minimum score for that domain is two. Takken et al. (2006) questioned the importance of these rules and explored the use of less complex score calculation methods [7]. Their results indicated that calculating the average of the thirty items improves sensitivity to change.

The main goal of this study was to evaluate the score distributions of the original [8] and the revised version of the Dutch CHAQ_Cat_. Furthermore, we wanted to explore the individual influence of different score calculation methods and the eight more challenging items as proposed by Lam et al. (2004) on these score distributions. We hypothesised 1: The revised CHAQ to have a more normal score distribution than the original CHAQ, 2: A less complex score calculation method to improve the score distribution of the original CHAQ, and 3: The eight more challenging items to have a positive influence on the score distributions of both questionnaires.

## Methods

### Questionnaires

The revised CHAQ with the categorical response option was translated and adapted following the absolutist approach with forward translations, consensus meetings, panel review, back translation, and, finally, authorisation by the developers [9].

**Figure 1:**
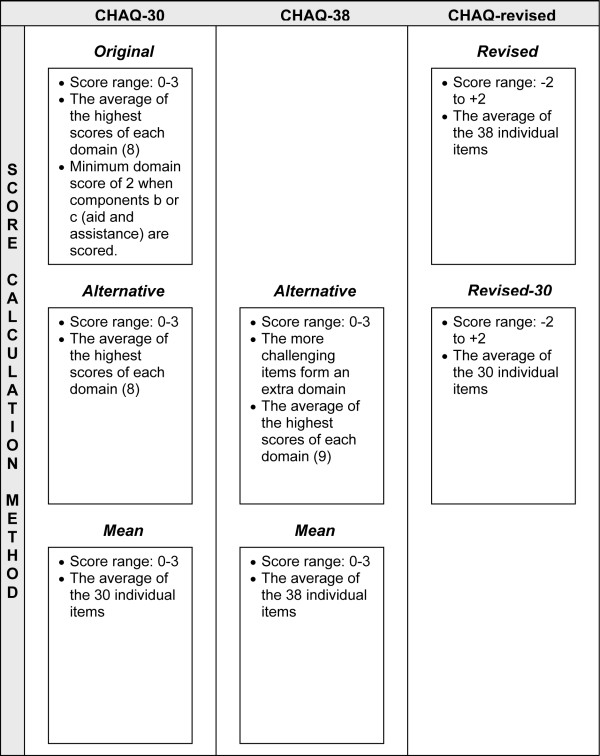
Score calculation methods of the seven different versions of the Childhood Health Assessment Questionnaire (CHAQ).

Varying with score calculation methods and the eight more challenging items resulted in seven different (score-) versions of the CHAQ (Figure 1).

### Participants

Two convenience samples of children with JIA were studied. As we were studying the ceiling of the CHAQ, patients with remitting disease were also included. Sample A included 59 children from a transition of care study in patients between 12 and 25 years of age. They completed the original CHAQ and the eight more challenging items. Sample B were 31 children who attended the outpatient physical therapy department of the Wilhelmina Children's Hospital (WKZ) and Medical Center Utrecht, for a regular check-up between May 2005 and December 2005. They completed the translated version of the revised CHAQ.

### Statistical analysis

Data were entered and analysed using SPSS 11.5 for Windows. To evaluate the score distributions of the seven (score-) versions of the CHAQ, box-and-whisker plots and the Kolmogorov-Smirnov (K-S) one-sample test of normality was used. Box-and-whisker plots give a visual representation of the median, the quartiles, and the smallest and greatest value in the distribution. The K-S test statistic represents the largest absolute difference between the observed distribution and theoretical cumulative distribution functions. A p-value less than .05, was considered statistically significant. To analyse the differences between the groups on anthropometric parameters a Student t test was used.

## Results

### Participants

The 59 children from sample A had a mean age of 14.85 (8–25) years and a mean duration of joint complaints of 8.30 (0–20) years. They scored a median of .25 on the original CHAQ with a score range of 0 to 2.86. The 31 patients from sample B had a mean age of 10.81 (4–18) years and a mean duration of joint complaints of 2.90 (0–11) years. They scored a median of -.22 on the revised CHAQ with a score range of -1.55 to +1.05. The characteristics of the participants from both samples are summarized in Table 1. Group B was significantly younger (p < .0000) and had significantly shorter disease (p < .0000) compared to group A.

**Table 1:** Participant characteristics of sample A and B.

	Sample A (n = 59; 18 ♂)	Sample B (n = 31; 16 ♀)
Age, mean (range)	14.85 (8–25)	10.81* (4–18)
Duration of joint complaints, mean (range)	8.34 (0–20)	2.90* (0–11)
Original CHAQ, median (range)	.25 (0–2.86)	-
Revised CHAQ, median (range)	-	-.22 (-1.55 – +1.05)

Sample A = patients from the transition of care study in patients with JIA; Sample B = patients who attended the outpatient physical therapy department of the Wilhelmina's Children Hospital and Medical Center Utrecht, for a regular check-up between May 2005 and December 2005. *) p < .0000

### Hypothesis 1

**Figure 2:**
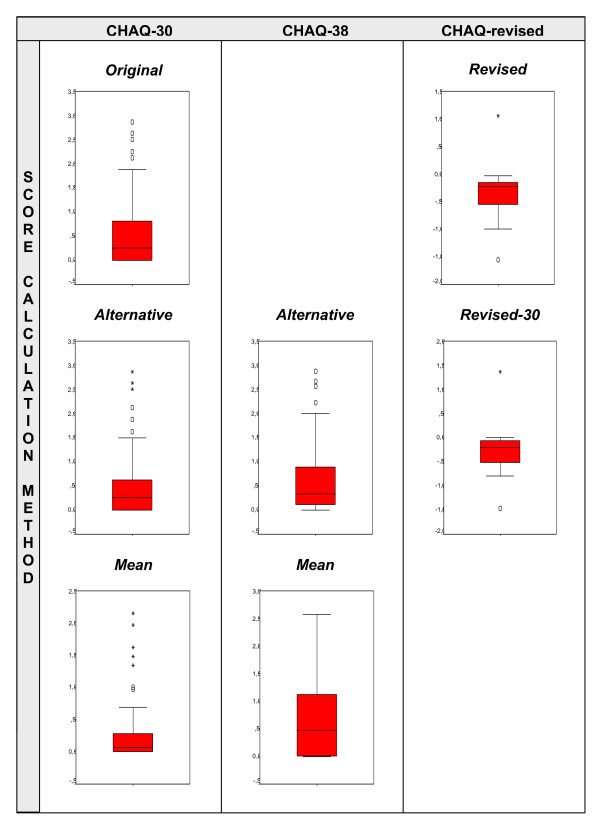
Box-and-whisker plots of the seven different versions of the Childhood Health Assessment Questionnaire (CHAQ) Score calculations as described in Fig 1.

As hypothesized, the box-and-whisker plot of the revised CHAQ shows a more normal score distribution than that of the original CHAQ (Figure 2). This is also reflected in the K-S one-sample test of normality, with a p-value of .008 for the original CHAQ and .136 (statistically significantly normal) for the revised CHAQ.

### Hypothesis 2

We also hypothesized that replacing the original score calculation method for a less complex one would improve the score distribution of the original CHAQ. The box-and-whisker plots in the first column of Figure 2 however show the opposite. After omitting the questions about aids and assistance (Alternative) the ceiling effect raised. The ceiling effect rose even further when the average scores of thirty items were calculated (Mean).

### Hypothesis 3

Finally, the eight more challenging items indeed have a positive influence on the score distributions of both questionnaires. The middle column of Figure 2 shows that adding the eight more demanding items to the Alternative and Mean version of the original CHAQ fully compensates for the raise in ceiling effect as seen in the first column. Removing the eight more challenging items from the revised CHAQ (Third column, Figure 2), shows a similar change in the score distribution as in the original CHAQ, raising the ceiling effect.

## Discussion

We have shown that the revised version of the Dutch CHAQ_Cat _shows a significantly normal distribution statistically compared to the original Dutch CHAQ. However, opposed to our hypothesis, a less complex score calculation method of the original CHAQ did not improve the score distribution. Thirdly, the eight more demanding items as proposed by Lam et al. (2004) did have a positive influence on the score distribution of the original CHAQ as well as the revised CHAQ.

Our results are in agreement with the findings of Lam et al. (2004), but also show a crucial difference. The score distribution of the revised CHAQ was significantly normal statistically, but the score range was very narrow. This limits the applicability of the questionnaire in clinical research and clinical settings, because of a decreased ability to detect changes over time. This difference could be explained by the homogeneous groups in this study with only JIA patients. Lam et al. (2004) included patients with JIA, as well as other rheumatic disorders, injuries, fractures, spina bifida, and hemophilia with a history of haemarthroses. A second possible explanation is seen in the cultural differences. Dutch children seem to underestimate their capabilities compared to Canadian children. Even though they could score between -2 and +2 (+1 and +2 perform better than peers) on the CHAQ_cat_, almost all respondents scored below 0.

Our results did not concur with Takken et al. (2006), who concluded that the original score calculation method of the CHAQ could be replaced with a less complex one without clinical and psychometrical consequences.

A limitation of this study was that the samples we used were convenience samples consisting of different patients. Sample A was an existing historical data-set of children from a transition of care study in children with JIA between 12 and 25 years of age, and the data of sample B were gathered retrospectively from patient files during January and February of 2006. Both samples did not complete the original as well as the revised CHAQ, but only one or the other (of the two). Therefore the observed differences could in part be caused by differences in patient characteristics, such as age and duration of joint complaints.

The results of this study confirm a ceiling effect using the original CHAQ-30 with either score calculation method. This emphasises the need for further exploration to improve the CHAQ. The original CHAQ-38, with the "mean" score calculation method as well as the revised CHAQ, show less ceiling and therefore are a better alternative for future studies in paediatric rheumatology. However, there is a need for further prospective studies to support our findings.

## Competing interests

The authors declare that they have no competing interests.

## Authors' contributions

JWO summarized the existing literature, prepared and analyzed data of samples A and B and prepared the draft of the manuscript. PhP offered the data set of sample A and supervised data analysis of this sample. TT and JN developed the methodology and supervised the statistical procedures. PJMH facilitated the logistics, the use of the two datasets and senior read the manuscript.
